# Trans(gender) journeys: rights and the (non-)recognition of “human”

**DOI:** 10.3389/fsoc.2023.1172471

**Published:** 2023-06-14

**Authors:** Liliana Rodrigues, Ana R. Pinho, Nuno Santos Carneiro, Conceição Nogueira

**Affiliations:** ^1^Center for Psychology at the University of Porto, Faculty of Psychology and Educational Sciences of University of Porto, Porto, Portugal; ^2^Institute of Social Services of Porto, Porto, Portugal

**Keywords:** critical psychology, human rights, (de)pathologization, trans, gender, biomedical model, transfeminism, intersectionality

## Abstract

A human right paradigm has been challenging the biomedical perspectives that tend to be normalized in the Western context concerning the lives of trans people. The aim of this study is to understand how trans people in Portugal and Brazil perceive the (non-)recognition of their socio-cultural, economic and political rights. Specifically, the study intends to know in what extent these perceptions influence the processes of identity (de)construction. For this purpose, 35 semi-structured interviews were conducted with people self-identified as trans, transsexuals and transvestites in Brazil and Portugal. The narratives of the participants were analyzed according to the thematic analysis method and the following six main themes emerged: (i) Who are the rights for; (ii) Types of rights; (iii) Paradigm of distribution of rights; (iv) Local or global rights; (v) Non-recognition of the “human”; and, (vi) Transphobias (and cissexism). The results allowed the knowledge of rights and the non-recognition of the “human” which is the central organizer of the analysis. Among the main conclusions of this study, we emphasize the circumscription of rights to certain international, regional and/or national contexts; the existence of local instead of global rights, since they are influenced by regional and international law, but they depend on the legislation in force in each country; and the way human rights can also be understood as a platform of invisibility and exclusion of other people. Based on a commitment to social transformation, this article also contributes to rethinking the violence that is exercised on trans people as a continuum, whether through ‘normalizing devices' by medical contexts, family contexts, public space, or even through internalized transphobia. Social structures produce and sustain transphobias and, simultaneously, are responsible for fighting them by changing the paradigm about the conception of transsexualities.

## Introduction

In Portugal, on February 22, 2006, in the city of Oporto, Gisberta Salce Junior, a Brazilian trans woman, was murdered. Gisberta was not only a trans woman; Gisberta was also a Brazilian immigrant, HIV-positive, drug addict, sex worker, and homeless. Her belonging to these social groups placed Gisberta in a situation of extreme vulnerability. Gisberta was tortured by a group of youths and later thrown into a well. The sentence mentions that the torture was “nothing more than a bad joke that ended badly”, neglecting the real motives of the aggressions: transphobia^1^ (Panteras Rosa, 2006; Oliveira, 2015).

In Portugal, on September 9, 2019, in the city of Almada, Lara Crespo, a Portuguese trans woman, committed suicide. Like Gisberta Salce Junior, Lara was not only a trans woman; Lara was also a 48-year-old woman in a precarious situation. She lived on the margins of society and she was constantly exposed to hate, therefore, she lived a life of extreme vulnerability (Rodrigues et al., 2021b).

Gisberta was murdered; Lara committed suicide (Panteras Rosa, 2006; Oliveira, 2015; Rodrigues et al., 2021b). Both were targets of the same violence: transphobia.^2^

In the 21st century, a new paradigm about trans people is emerging: a human rights project. So far, the dominant focus was the medical-psychological perspective, which defines trans people as “deviant” from the sex/gender binary norm. This biomedical perspective tends to be naturalized in the Western context through different ideological agents, but it has been challenged by the new approach that focuses its attention on the legal and social situation of trans people, highlighting the human rights violations to which they are subjected (UN, 2008; Hammarberg, 2010; Pillay, 2013; Platero, 2014; Romboli, 2021; Hidalgo, 2022). The emergence of the new paradigm arises from the evidences of a systematic violence and discrimination directed at people on the basis of their gender non-conforming identity, which ranges from discrimination in employment, access to health, education, family, public space, to physical and sexual assaults, torture and homicide (UN, 2008; Hammarberg, 2010; Sennott, 2011; Pillay, 2013; TGEU, 2015a,b, 2021; Rodrigues et al., 2021a).

The new approach perceive the pathologization of transsexualities as a way of stigmatization with harmful consequences for trans people that stem from stigmatization processes (Sennott, 2011; Missé, 2014). In this context, fundamental human rights are not recognized and trans people cannot exercised them. Several rights are not assured. The international human rights regime afirms that protection right is for all people. But the right to life, personal security and privacy; the right to be free from torture, arbitrary arrest and detention; the right to be free from discrimination; the right to freedom of expression, assembly and peaceful association (UN, 2008; Pillay, 2013; Rodrigues et al., 2021a); and the right to autonomy of their identities and management of their bodies (Suess, 2010, 2011; Missé, 2014; Platero, 2014) are not a reality for trans people yet. It is, therefore, with the aim of guaranteeing trans people the full exercise of these rights that this perspective emerges.

The legislation of most member states of the Council of Europe does not explicitly recognize transphobia as a possible motive for hate crimes: the Scottish law was the first to include transphobia in the typologies of hate crimes. As a result, in most European countries, trans people have been excluded from specific legal protection, despite the high risk of becoming victims of hate crimes (Whittle, 2006; Costa et al., 2010; Hammarberg, 2010; Jesus, 2012). For example, transphobia was not considered as an aggravating factor in hate crimes against trans people in the sentencing of perpetrators of hate-motivated homicides in Portugal and Turkey (Turner et al., 2009; Hammarberg, 2010).

Besides the protection from transphobia, the recognition of legal identity of trans people also depends on the country and the region of the world to which that country belongs (Pearce, 2018; Hidalgo, 2022). Following the same conceptual line defended by Butler (1999, 2004), Coll-Planas (2010), and Pearce (2018) understands the pathologization of trans identities as a form of gender violence, as well as a form of transphobia that is exercised by the state and by medical institutions that intend to “cure” trans people. It is framed in this scenario that some states have not legitimized trans identities, contributing to the violation of their fundamental human rights.

In Portugal, Law No. 7/2011^3^ of 15th March allowed the name and sex change in the civil register - a procedure in which was charged a fee of 200€ under the terms of Article 18°6.12 of the Regulation of Emoluments of Registries and Notaries, Decree-Law No. 322-A/2001 of 14th December, then revoked and an approval of exemption fees for the sex and respective change in the civil registry in Portugal was adopted in 1st April of 2020^4^ - Law No. 19/2013 of 21st February made an amendment to the Criminal Code, including transphobia as an aggravating factor in hate crimes,^5^ and Law No. 28/2015 of 14th April establish the right to equality in access to employment. All these laws contributed to the recognition and legal protection of transsexualities. In 2018 is approved the Law No. 38/2018 of 7th August that allowed the right to self-determination of gender identity and gender expression as well the protection of the sexual characteristics of each person,^6^ removing the mandatory diagnosis for trans people. In 2019, it was also approved the Order No. 7247/2019^7^ of the Presidency of the Council of Ministers and Education—Offices of the Secretary of State for Citizenship and Equality and the Secretary of State for Education—which establishes the administrative measures for implementing the provisions in Article 12 No. 1 of Law No. 38/2018 of 7th August.

In Brazil, in 2013, Ordinance No. 2,803 dated on November 19, 2013,^8^ redefines and expands the “transsexualizing process” in SUS (Unified Health System). The diploma mentions the integration of care for transsexuals and transvestites, preventing the restriction or the centralization of therapeutic goal to sexual reassignment surgeries; the interdisciplinary work; the humanized reception without discrimination; and the respect for differences and human dignity at all levels. Also, through the Ordinance No. 73 dated on June 28, 2018,^9^ which allows transgender people to change prename and gender in birth and marriage records in the Civil Registry of Natural Persons, it was possible to remove the diagnosis requirement for trans people to have their identities recognized in Brazil.

In the light of the above and considering that some countries have made a commitment in the international domain to combat discrimination based on gender identity, there are still many legal and political gaps. Therefore, it is essential to adopt an intercultural dialogue and to discuss the rights of transgender people—both at national/regional and international level—involving international organizations, national human rights institutions, non-governmental organizations, academia, media professionals, etc. Moreover, it is important to embrace socio-political stances committed to a critical human rights perspective, to enhance the living conditions of trans people (Piñeroba, 2008; Rodrigues et al., 2021a) and to affirm the free expression of gender identity, without discrimination, as an inalienable human right (Arán and Murta, 2009; Suess, 2010). Only in this way, it will be possible to build alternatives of resistance and humanizing recognition of these people and for them (Santos, 2009).

It is important to reread human rights from alternative locations, from the zones of exclusion or from the perspectives of the excluded subjects. Focusing on excluded people and their stories can bring the human rights project back to a “new” space of meanings, revitalizing the political and ethical action of human rights construction (Kapur, 2006; Mullally, 2009; Hidalgo, 2022).

In several places around the world, many activists and non-governmental organizations have fought for human rights of these oppressed groups, developing anti-hegemonic human rights discourses and practices, proposing non-universal conceptions of rights and intercultural dialogues (Santos, 1997). Some groups have used the human rights platform as a tool to recognize their rights, assuming the importance of perceiving trans people from a human rights perspective. Nonetheless, it is important to critically consider the non-historical and universalizing character of the more traditional perspective of human rights because human rights can also be a platform of exclusion (Madson, 2022).

The adoption of a critical reflection (Kapur, 2006) on the mainstream conception of human rights it will allow us to recognize, even in if temporarily, that it can be maintained through knowledge shared with other cultures and societies (Santos, 1997; Schritzmeyer, 2008).

These critical proposals favor the construction of a society in which the differences and singularities of people are discussed and contemplated, and in which the different axes of social identity (e.g., gender, sexuality, age, class, nationality, etc.) are articulated. This implies new challenges for the effective application of principles such as equality, social justice, and societal democracy (Santos, 2009), as well as the transformation of the conception and practice of human rights into a cosmopolitan project that transcends globalized localism (Santos, 1997; Hidalgo, 2022).

## Methods

### Participants

In this study 35 people self-identified as transsexuals, transvestites^10^ and trans were interviewed. The designations used were mentioned by the people themselves and the participants' biographical data resulted from an analytical process, which was shaped by the research paradigms: constructionist, feminist and intersectional. The constructionist paradigm defends that critical psychology is opposed to the position that science is impartial, non-political and value-free. Its assumption settles in the deconstruction of social categories, with the goal of promoting social justice, the wellbeing of communities in general and oppressed groups in particular (Prilleltensky and Fox, 1997; Parker, 1998). The feminist and intersectional paradigm is a political movement that has contributed to the deconstruction of gender binarism and essentialist perspectives by reinforcing the intersectional character of oppressions. It values the political struggles and personal experiences of trans people and it is not restricted to those who wants to participate in it, thus enabling it to actively involve both people who identify as trans and people who identify as cis (Jesus and Alves, 2010; Rodrigues, 2016).

All participants presented discourses of a non-conformity between the sex designated in the birth register and the gender to which they feel they belong. Twenty-one persons self-identified as female and fourteen as male, with ages ranging from 16 to 55 (M = 30.17 and SD = 8, 75). Twenty-four people were Brazilian and eleven were Portuguese. At the time of the interviews thirty people were single, three were married, and two were divorced. Twenty-one people self-identified as heterosexual, five people as bisexual, one person as gay, two people as lesbian, one person said he/she was not physically, psychologically or emotionally attracted to anyone, four people said they were attracted to people, and one person said he/she did not know. As for educational qualification, twenty-one people had completed high school, twelve had completed higher education, and two people had completed elementary school. Finally, regarding professional status, eleven people were employed, four people were unemployed, and twenty people were in other professional situations (e.g., precarious jobs, no employment contracts, research fellowship, and sex work).

To protect the identification of the participants of the study, a code was created to identify the characteristics of these subjects. The identification code for a subject begins with the interview number, followed by the initial of the name, the gender and finally the initials of the country. Here, are two examples of coding for each country: the code “17-B.M.BR” corresponds to the interview 17, the initial of the name is B, the gender is male and the person is from Brazil. The code “1-A.F.PT” corresponds to the interview 1, the initial of the name is A, the gender is female and the person is from Portugal. For further guarantee of confidentiality and anonymity of participants, the letters chosen for the initials of the names are random and, therefore, do not coincide with the names with whom people identify themselves.

### Instrument of data collection

To data collection, a semi-structured interview script was used. Due the different historical, sociocultural, economic and political contexts in Portugal and Brazil, the same interview script had two versions (the version applied to people in Portugal and the version applied to people in Brazil). The two versions of the interview script were built after an in deep literature review on trans issues.

This script was divided into three parts: the first referred to the informed consent, where the participant read the conditions of participation in the study; the second was related to the interview itself, which included semi-structured questions that allowed answering the purpose of this research; and, finally, the third corresponded to the collection of the participant's biographical data.

Throughout the interview process, the script served as general guidelines for the interviews only and a flexible posture was adopted, according to the interviewee and the countries (i.e., Brazil and Portugal).

### Data collection procedure

There was a prior contact with some participants before the beginning of the study, therefore, the data collection process was intentional. The invitation for the interviews and subsequent data collection had two phases: it started in Brazil from October 2013 to January 2014, and it ended in Portugal from March 2014 to October 2014.

The interviews were conducted in public places defined by the interviewees with the guarantee of adequate conditions to collect audio records. The interviews had an average duration of 60 min. After the full transcription of each interview, data analysis followed. The NVivo 8.0 software was used to organize the interview material, mainly due to the volume of material collected.

### Data analysis procedure

Thematic analysis is a method widely used in qualitative data analysis and it aims to identify, analyze, and report patterns (themes) in the data, enhancing the understanding of explicit and implicit meanings associated with textual data (Braun and Clarke, 2006).

In this study, a constructionist, feminist, and intersectional paradigm was adopted in the thematic analysis of the data. One of the strengths of this type of research, assuming a constructionist paradigm, is that the researcher, rather than being responsible, is implicated in the entire research process (Prilleltensky and Fox, 1997; Parker, 1998; Rodrigues, 2016).

The data analysis followed the recommendations proposed by Braun and Clarke (2006, 2013), which includes six steps: (i) familiarization with the data; (ii) codes generation; (iii) themes searching; (iv) themes revision; (v) themes definition and naming; and, (vi) report production. This analysis was mainly deductive (theoretical) because the literature review on the topic informed the practice. However, some topics emerged from the data, thus, bringing an inductive character that strengthened the analysis. Therefore, in a first phase the themes were semantic and in a second phase of analysis were more latent. It was possible to identify more latent themes from the participants' narratives due to the activism involvement of the first author that allowed a better understanding of the historical, sociocultural, economic and political contexts in Portugal and Brazil. Also, the detailed readings on the theme in both countries prior to the application of the interviews and the researchers' privileged contact with diverse contexts that allowed a proximity to the concrete lives of these people made it possible for a deeper knowledge of participants' speeches in the context in which they were produced.

## Data analysis

In this section it will be presented the analysis that emerged from the data and that was shaped by the constructionist, feminist and intersectional paradigm. From the analysis the following six themes, which are interrelated, were identified: (i) Who are the rights for; (ii) Types of rights; (iii) Paradigm of distribution of rights; (iv) Local or global rights; (v) Non-recognition of the “human”; and, (vi) Transphobias (and cissexism).

The central organizer of the analysis was designated “rights and (non-)recognition of the ‘human”'.

Although with a critical perspective to the human rights platform, data showed that some trans people use it as a way to access and have their rights recognized. Also, it was possible to observe the contexts, types of transphobias (and cissexism), and other “isms” as some of the ways in which the “human” is not recognized.

Due to the extensive volume of collected material, the schemas of each theme are presented; it is also presented the themes, the codes and the most illustrative extracts. The purpose of this analysis is not only to describe the data, but to problematize it in relation to the research questions/goals of the present study. This interpretation and discussion take place in a dialogical process with the literature on the subject.

The themes that emerged from the data will be analyzed and discussed in more detail below.

### Who are the rights for

Here, three codes emerged from data: “rights for all”; “rights for people/individuals”; and “rights for groups/collectives”, which allowed the identification of the theme “who are the rights for”.

Most people mentioned that rights should be for everyone, however, they do not cover all groups/collectives, nor all people. The following examples illustrate the code “rights for all”:

“*It's hard… I had never thought about that* [who is protected by the human rights platform]. *I think it must be both* [people/individuals and groups/collectives]. *I think it should be both. At the same time, it is individualized when we are thinking about a human subject, we are also thinking about groups that are excluded in some way, that have their humanity denied, and then resort to human rights. I think it is both”* (21-L.M.BR).

“*Well, I think that if it does not work for everybody it should. From the perspective of what is understood by human rights (...)”* (11-E.M.PT).

Fewer people mentioned that rights are for people/individuals and other participants referred that they are for collectives. Here are some examples, respectively:

“*For people, I do not think it makes so much sense to give rights to a collective, an association or whatever. In principle, these collectives and associations work or have people behind them, so in my opinion it is always rights for the people. For the collectives and associations, it does not matter because associations and collectives always have ideas behind them and it is not the ideas that deserve rights but the people that deserve rights regardless of the ideas they have”* (7-I.M.PT).

“*It works for collectives; in my personal case it does not serve me much. It does not suit me very specifically”* (9-J.M.PT).

Despite the recognition that rights should be for everyone, there is a conception that they are limited to some people or groups (collectives) (Santos, 1997, 2009; Romboli, 2021). Moreover, this analysis allowed us to question the place of oppressed people and groups in the human rights platform and how much some people and groups do not feel recognized in this construction of the “human” (Santos, 1997, 2009). This construction of human, and consequently of subjects with rights, is based on an abstract (and universal) perspective, which focus on the image of white, heterosexual man and neglect other (oppressed) groups (Rodrigues et al., 2021a), as it was observed, for example, in the historical women's suffrage movement (Waite, 1887). On one hand, this construction of an abstract subject, contributed to the non-recognition of the “human” to women, and on the other hand, also, contributed to erase other groups, such as lesbians, gays, bisexuals, and especially transgender people.

### Types of rights

Two other codes—“right to self-determination of bodies and identities” and “right to health”—were made to emerged from data, which lead to the identification of the theme “types of rights” that are (or should be) recognized for trans people. From the data it was possible to identify two fundamental rights for trans people, which are not specific to this population, but they add substantial vulnerabilities and obstacles when they are not guaranteed to them. Both rights are related to each other.

Most of the participants mention the right to self-determination of their bodies and identities (but that it is not always guaranteed). The non-recognition of this right adds vulnerability to their condition. They mention that this right is central to people and should not be controlled by doctors and/or judges, who are sometimes involved in the life contexts of trans people. In the collected data emerged specifically the right of trans men to become pregnant as a right to self-determination of bodies and identities. Trans men are recognized as having the right to alter their bodies while maintaining the legitimacy of their identity. According to participants, the recognition of the right to identity cannot be limited to requirements. In opposition, there are some countries that require a person to be single or divorced, in order to change his or her name and gender in the civil register.

In addition to recognizing their right to self-determination of bodies and identities, the people interviewed also report progresses concerning this right, although with advances and setbacks. Here are some examples:

“*Psychiatrists and psychologists do not know, nor there is any way they can judge a person's gender identity. (...) There are no tests, there is nothing we can do to know. Then, how are we going to do this? (...) For example, I do not do a surgery to be a woman, but I do a surgery because I am a woman”* (2-C.F.PT).

“*Transgender men getting pregnant have always existed since transgender men exist, and they will continue to exist if these men wish. We are not going to stop having a pregnant man just because we force him to say he is a woman on a civil registration (...)”* (3-T.F.PT).

Data shows some social misconceptions regarding pregnant men, as in some literature on the area. If a trans man, for example, decides to use his reproductive organs to become pregnant he is just taking advantage and valuing the characteristics of his body as other people do (Zinkunegi, 2013; Platero, 2014). At no time this behavior should delegitimize any identity. A trans man who wants to become pregnant is not being less of a man because of that, he is just reflecting human diversity.

Another type of rights that emerged from the data was the “right to health.” This code is strictly linked to the right to self-determination of bodies and identities, because some trans people, who wish to alter their bodies to conform with their identity, need to access health care. Here are some examples:

“*(...) but in the case of transsexuality all those that are inherent to transsexuality, hormone therapy, surgeries whatever they are… it is a principle that we have in the Brazilian State, health is a right of the population and duty of the State. Based on this, any person has the right to demand anything. Of course it is not that easy, you need a legal fight, but you end up winning, because it is the principle of the Brazilian State that health is a right of the population and a duty of the State”* (23-L.F.BR).

“*Everyone has the right to health, without needing any mechanism to validate obligation. The right to health cannot be conditioned to anything”* (3-F.F.BR).

### Paradigm of distribution of rights

Two more codes emerged from data: “equal rights for all” and “social justice”, which allowed the identification of the theme “paradigm of distribution of rights”. From these codes it is possible to understand how trans people recognized the distribution of rights: based on an egalitarian positioning, without attending to the specificities of the groups; or, on a fair positioning, attending to the particularities of the groups.

When the paradigm of distribution of rights was based on social justice, people mentioned that it should be possible to give “more” rights to people who belong to historically oppressed groups, enabling them to access things that otherwise would be more difficult. Participants provided some examples of actions based on the principles of social justice, also coined affirmative actions, as is the case of quotas in Brazilian public higher education for black (non-white) people. The present study will not focus on this matter, however, it is important to note that these affirmative action measures are not an end in themselves, but a means to an end. It is about restoring social justice for historically oppressed black or non-white people (Kymlicka, 1995, 2001).

Fifteen participants mentioned that the distribution of rights should be equal for all people, in the sense of enforcing the rights of the person, for example, to access civil marriage (if they so desire); to adoption; to the right to health care; to the right to housing, among others. According to these participants, rights should be for all people, regardless of their sexual orientation, gender identity, economic or social status. Here are some examples:

“*I believe, it is as I said, if it is to live in a society the same right that I have you also have to have, because you live in the same society as me”* (4-D.F.BR).

“*Rights should be…, should be in the first place for everyone. Everybody should have the same rights, whatever they are: education, health, food, work, everything. Do you understand? So, rights on a general level should work for everything. (...) I think they should be equal for all people. Human rights should be equal for all people. As it is in our constitution, regardless of creed, religion, race, sexual orientation, and gender identity which is not mentioned there but it should be”* (1-A.F.PT).

Another fifteen participants mentioned that the paradigm of distribution of rights should be based on social justice. In other words, it should exist rights for all people considering their specificities, in order to eradicate the historical oppression of oppressed groups. Here are some examples:

“*So, I believe that yes… there should be equal rights and there should be no exceptions… the black person should not be treated differently; the transvestite should not be treated differently, you know? But since we do not have this, then, we have to make sure that some receive a specific benefit, so that they can... People talk like this: slavery has already been abolished, but when the blacks were inserted into society, they were not inserted in a fair way (...)”* (15-E.F.BR).

“*It is not enough for us to say that rights have to be equal for everyone if society does not treat everyone as equals (...) That idea of false symmetry, that we are all equal before the law, is fallacious. If we are not equal, if some people need other rights they have to be granted (...)”* (3-F.F.BR).

### Local or global rights

Here, two codes emerged from data: “human rights as a platform to fight and to protect rights”, and “recognition of rights circumscribed to states”. These made it possible to identify the theme “local or global rights”, which allows us to problematize the construction of human rights as universal (global) or limited to certain contexts, regions and/or countries where they are applied.

Twenty-seven people mentioned that they recognize the human rights platform as a tool to fight for and protect their rights. Although trans rights recognition has not always occurred, the human rights platform is seen, by the participants of the study, as one of the possibilities to achieve that. The possibility of a paradigm shift to recognize trans people as human, rejecting the notion of pathology, was also considered. The following are some examples:

“*For me human rights are… are completely related to the right to exist and to be respected within your living, whatever it is. To be respected and to have access to all guarantees and to fully exercise citizenship. And to be respected in the sense of physical, emotional integrity and everything else”* (20-C.M.BR).

“*It does because it is the way you fight. It is the way society has arranged itself, how society is organized is the way it fights. I talked about the human rights issue… the way by which you get some kind of humanity is through human rights. That's the game we play today. I don't know if it is good or bad, but it is what we have today (laughs). It is what is there, so it makes sense because that is how it is organized (...)”* (3-F.F.BR).

Another code that emerged from the data was “recognition of rights limited to the states”. Some participants mentioned that the recognition of rights has been limited to certain international, regional and/or national contexts. Thus, they stated that rights are local and not global and that they are dependent on the legislation in force in each country and influenced by regional and international law. Narratives of participants enlightened that, depending on the countries, this recognition is different. In Portugal, there is still pressure from the European Commission on Human Rights, the UN (United Nations) High Commission on Human Rights, to recognize the rights of people in each member state. In Brazil this pressure continues to be made at the regional level by the OAS (Organization of American States) and in international terms also by the UN High Commissioner for Human Rights. These different types of influences can explain the different impacts on national legislation in each of the countries. In Brazil there is legislation in federal terms and State resolutions. Here are some examples:

“*(...) human rights are very fragmented. For example: what I have here is different from what the girl in the North has* [region of Brazil]. *Do you understand? For example, I run less risk of being murdered for being a transvestite and transsexual in this case, a trans woman* [for living in the South of Brazil] *than another girl in my same condition there in the Northeast* [region of Brazil]” (15-E.F.BR).

“*There are countries, for example talking specifically about transsexuality… is not accepted in all countries. There are countries where you get arrested for being homosexual. There are countries where you cannot commit adultery, because otherwise you are also arrested and so on”* (10-B.M.PT).

### Non-recognition of the “human”

From the data, the following codes also emerged: “questioning the social structure for the recognition of rights”; “human rights as a platform of invisibility and exclusion of other people”; and “no access to health and its implications”, which allowed the identification of the theme “non-recognition of the human”.

In the code “questioning the social structure for the recognition of rights” some participants problematized the social structure for the recognition of rights as not being able to attend to the effective recognition of people's rights. Some participants referred that this recognition is limited to the States. Despite the acknowledgment that the Universal Declaration of Human Rights (with the construction of its economic and social covenants) has enabled the guarantee of rights for some people, they also mentioned that this guarantee is not effective for everyone.

On the one hand, if conceiving the idea of universality of rights may be useful to problematize that all people should be considered human and, therefore, have access to basic rights, on the other hand, thinking about rights in a universal way, without problematizing the Western construction of the human rights discourse, may generate a limited discussion (Santos, 1997). Here are some examples:

“*Law for me is very glued to State, to Federation, to the idea of disciplining the other and of punishing. This pun does not stick, it does not stick with people. It does not create respect, I do not know if it is a problem of Brazil, I think it is a problem of the world, relationships are made this way”* (1-P.F.BR).

“*Human rights are beautiful to see, difficult to achieve. It is a category that I think… it is beautiful to talk about human rights, but I think it is kind of a mechanism created even in a capitalist logic so we can think there is a light at the end of the tunnel to end inequalities, but in reality, in practice, we see that human rights are pierced by corporativist logics and by many other issues… if these other issues are not in conformity, human rights do not work”* (3-F.F.BR).

Another code that emerged from the data was what we called “human rights as a platform for invisibility and exclusion of other people.” This code is prevalent throughout the analysis and can be read in the narratives of different people. Most participants, while recognizing that the human rights platform still serves as a tool to fight for and protect rights, also report that it continues to neglect and exclude groups that have not been named, that have not been recognized concretely as having rights. Here are some examples:

“*(...) we get into these political and demagogic issues of the right to life, right to come and go, survival and such, but we know that this is violated. My own right is violated. My own right to be a woman is violated. So, this is more or less what I understand by human rights (...). When we talk about this issue, for example, the right to life, we never think about, for example, black women from the slums. Because the black woman from the slums in Brazil is not reached. In other words, the same right to life for one is not the same right to life for another. The number of deaths of young black men that die every month in Brazil is like a plane crash but it is not the same right to life “* (15-E.F.BR).

Another code that emerged from the data was “no access to health and its implications”. Several participants mentioned they do not have effective access to health care when it is under the control of health professionals. They also referred the two main implications of not having access to health care services for their lives: one of them is self-medication and another is access to clandestine services. Here are some examples:

“*I use to block the male hormone. And I use a very low level of estradiol to not let the testosterone levels increase. Self-medication, without monitoring, because my parents do not accept it, but I also do not want to become masculine, do you understand? If I did not interrupt puberty, if I had not interrupted it, I wouldn't be where I am today. It would be much harder for me. I started at 16 without anyone knowing”* (19-R.F.BR).

“*(...) the vast majority* [of people] *start irregularly, let's say, because it is very difficult to get professionals that know how to deal with the cause”* (17-B.M.BR).

### Transphobias (and cissexism)

Finally, the codes “transphobia for the ‘other”', “internalized transphobia”, “transphobias in context”, “other ‘isms”', “impacts of transphobias”, “transphobias as stigma” and “strategies to eradicate transphobias” emerged from the data, which allowed us to identify the theme “transphobias (cissexism)”.

Transphobias were transversal in the narratives of the participants of this study, as another form of non-recognition of the human. There are several dimensions of transphobias to be considered: who carries them out—whether the “other” or the person him/herself; in which contexts; what are their impacts on the lives of trans people; and, how to eradicate them.

The code “transphobia by the ‘other”' focus on how people feel and experience situations of transphobic violence perpetrated by other people. These situations of violence are motivated by hatred of trans people. This hatred is anchored in the oppressive system that devalues trans people and overvalues cis people.

Many of the participants reported transphobia experiences, perpetrated by other people, which reproduce the cissexist system of (Western) societies. The action of not recognizing the person's name is also a form of transphobia. Here are some examples:

“*Because I'm trans, let me see... I think so… when I went to get married, on my wedding day, I was with J. at the registry office and then the guy raped me several times by calling me by my civil name. He saw that I was not dressed as him. So, I take this as violence… a violence that he did to me”* (15-E.F.BR).

“*So trans people suffer a lot from that and I do not escape the rule. And I think if you ask ‘What is more important to you right now? To do the surgery or to change the name and gender? all trans people will be unanimous in their answer. They will all say it is the change of the name and gender because the surgery is something that is for you, but name and gender is something that is public, something that can make you vulnerable to some kind of prejudice (...)”* (23-L.F.BR).

Another type of transphobia that emerged from the data was “internalized transphobia.” The term “internalized transphobia” was used as a parallel to internalized homophobia. This term does not intend to blame trans individual and take out the responsibilities from society, but the opposite. It intends to dismantle the system that oppresses trans people, cissexism, also known as transphobia. The devaluation and prejudice about trans people embodied by the trans people themselves is therefore entitled internalized transphobia (Creighton and Kivel, 1992; Lewis and Arnold, 1998; Missé, 2014; Platero, 2014).

Some of participants developed discourses of internalized transphobia based on a latent analysis, by incorporating the devaluation of being trans or the overvaluation of being cis (Cabezas et al., 2013; Platero, 2014). Some trans people have demonstrated some situations of satisfaction by not being perceived as trans/simulating cisgender in some contexts. Despite recognizing the legitimacy of this contentment, since in some situations not being read as trans reduces the possibility of violence and stigmatization, it becomes crucial to problematize the oppressive system that sustains these discourses (Sennott, 2011; Missé, 2014). The following are some examples:

“*Until I was 23 I discriminated myself, I thought I was the biggest freak in the world. I thought I had no more solutions. I really was like a dead person (...). I think all my life I did not accept myself. Even today I do not accept myself completely. This acceptance is very difficult, because nothing corresponds to your mind. Your body does not correspond to what you would like it to be (...)”* (5-V.M.BR).

“*I go to a store, people treat me well and they tell me: oh, if you did not tell me you were transsexual I wouldn't even noticed. Because now I also do laser hair removal on my face and I went to talk to this girl and she said if I had not told her I was transgender, she wouldn't notice it. And I have had good experiences”* (5-G.F.PT).

Several contexts of transphobia were present in the data, such as: transphobia in health; transphobia at work; transphobia in education; transphobia in the family; transphobia generalized to various contexts; transphobia in love relationships; transphobia in the LGB(T) movement; transphobia in religion; and transphobia in public space (streets, bars, bathrooms). From this data the most frequent contexts of occurrence of transphobia were transphobia in health; transphobia at work; transphobia in education; and, transphobia in public space, especially in the use of bathrooms. Here are some examples:

“*(...) the only time I went to a health service to ask for care I was classified… the guard… the security person, at the time of distributing the tickets, he said ‘people get in line here and transvestites here'. In a public place”* (13-D.F.BR).

“*For me it was difficult, because when I was in that period of transition, I worked in a company and I was starting my process of taking hormones and they came and talked to me. They said it would be interesting if I did not change abruptly; that I should change little by little, not to shock people and so they would not have to intervene. That is what happened. I went slowly”* (2-M.F.BR).

“*(...) from 10 to 12 I suffered bullying at school, I was in the fifth grade, the big boys (...) wanted to beat me. Then I had to run to the school office until the bell rang to enter the classroom. Then I changed schools, I suffered bullying, but it was only behind my back”* (19-R.F.BR).

“*(...) the old daily issue of bathrooms. I practice sport, I practice karate for years (...) and until very recently my training partners knew me as a girl, about a month ago, (...) I finally assumed myself to my training partners as a man and the question arose ‘ok, you are now a man, but which shower room do you go to? Where do you go to change your clothes at the beginning and at the end of the training sessions?' I was like, ‘yeah, I don't know how to answer that', so what happened? Now, when I have training, there is the men's locker room, there is the women's locker room, and there is a separate bathroom that I go to, and I don't really know. Is that discrimination? I feel that it is, but at the time I was the one who proposed this solution, because I do not feel comfortable yet, in a men's locker room, but I do not feel comfortable at all in a women's locker room either. So, it's like: either I stop going to the trainings, or I come with my clothes on from home, or I find another solution that puts me a little further apart, but I have to, so I ended up going that way (...). But, in that sense I feel that it is a bit of discrimination, but it is a discrimination that I cannot get around. I do not see a solution”* (7-I.M.PT).

As some studies report (e.g., Zucker and Bradley, 1995; Cohen-Kettenis et al., 2003; Roberts et al., 2012; Platero, 2014), gender variant or transgender children and youth are more vulnerable to rejection and oppression in societies where the cissexist and transphobic system operates, especially in school and family contexts, which control non-normative behaviors. This control does not occur only by teachers, fathers and mothers. Also, children and young people in school contexts surveille the gendered behaviors of their peers (Pereira, 2012) and when this behavior breaks with gender norms, the person is discriminated and abused.

Indeed, as Platero (2014) states, by focusing on the relationships between trans people and their contexts, we must problematize and transform the social structures that try to “discipline” people's identities. This work involves recognizing the responsibility of these structures to combat discrimination and to erase the view of transsexualities or gender variants as problems. The commitment to eradicating transphobia from social structures, such as schools, work and health contexts, along with other contexts in people's lives, such as family contexts, implies a set of strategies to fight oppression and protect people.

Some participants mentioned other discriminations they have experienced besides transphobia, such as racism, heterosexism and sexism. This code has been defined as “other isms”. Here are some examples:

“*(...) nowadays you have color prejudice, religion prejudice, gay as well, so if you had equal rights… but it's not like that. You have discrimination even in a store (...)”* (11-G.F.BR).

“*(...) some guys cornered me, saying that they were going to teach me what it was to be a real woman. What was it to be a real woman. I wore men's clothes and my neighbors believed that I was a lesbian, and then my father was afraid. And that is why my father was afraid: either you decide one thing or you decide another, but you cannot stay in the middle. And so, in my head, I had already decided that I would be male (...)”* (15-E.F.BR).

Another code that emerged from the data was: “impacts of transphobias”. Some participants mentioned, in addition to the discomfort that stems from these experiences of discrimination, lower average life expectancy, lower job opportunities, health problems, invisibility of their lives, difficulties for being trans, suicide attempts, prostitution, social denial and “normative integration” of the body. Here are some examples:

“*You know that our life is shorter than others in society. We face many risks, don't we? Illness, assault, or murder, so we try to make the most of it”* (12-F.F.BR).

“*With 15 I had a suicide attempt. Then it seems that my head got crazy, that person that was quiet, calm and used to stay in the corner, went crazy. I didn't sleep properly anymore. I started sleeping 2 to 3 hours a day and spent most of the time outdoors. When I felt like going to the beach, I would go to the beach. When I felt like doing something, I would go do something. I did not have any more… and I was growing up, this psychological question was here, it was always a question that I put off. I do not know what it is, I do not know what I feel. And from there I only went to see this psychological issue when I was 24, because I attempted a second suicide when I was 24”* (14-J.F.BR).

Several are the impacts of transphobia on trans people, or on those who break with gender norms. These impacts are marked, from an early age, when gender non-conforming children and young people incorporate the discourse of society, which are present in educational and family contexts. They reproduce discourses that make them believe what they do is bad, and, therefore, they are bad people or have a mental illness. This kind of discourse generates suffering in these young people (Platero, 2014).

In addition, they live less. Most of them do not grow old. These data corroborate the latest research on transphobic crimes in the world, with Brazil being considered the country in the world where there are more murders of trans people (TGEU, 2015a, 2021). As they age, they are faced an increasing vulnerability in their lives, especially because in addition of being trans and precarious people they are also elderly (Witten, 2004; Fernández-Rouco et al., 2012; Lopes, 2015).

As noted from the data, one of the impacts of transphobia was prostitution, however the data shows that not all trans people are prostitutes or are sex workers. However, there is still the stigma of prostitution associated with trans people, as well as the stigma of being HIV AIDS carriers. Here are some examples:

“*It's not a question of undervaluing prostitution in any way, but it was because I saw that I could not do it anymore, much because of the question of age. It is already 30 years old and even because of the question of vulnerability, of you being on the street and any person passing by, anything can happen and you are subject to everything there. So I chose not to have this for me anymore (...) everyone automatically put everything in boxes, transvestite or transsexual equals to prostitute, equals to money, equals to sex symbol, and less of a person*, [less of a] *citizen*, [less of a] *human being”* (4-D.F.BR).

“*(...) my life has been a succession of wrong facts, wrong paths that I chose by not knowing where to go and what to do. I ended up getting HIV last year (...). It could have been easier, much easier, you know* (crying and prolonged pause)*, like my HIV, I did not need to have that, you know. My HIV is a result of inconsequential relationships that I had, you know, emotionally, with men for not understanding what my position was in affective, sexual relationships, you know? So, I see it as a consequence of that, but it was necessary, because I think that only after everything I went through, I am ready to be the man I should have always been”* (17-B.M.BR).

Another code that also emerged was “strategies to eradicate transphobias”. Some participants pointed out some of these strategies, such as: transfeminism activism; contact with trans people; building conceptions of desirable trans bodies; and, (in)formation. Some examples are:

“*Ah the transfeminism. A lot of activisms in the vein is one thing that made me empower myself… Empower my body and feel more comfortable. I realize that I am a woman regardless of the genital that I have, I like my genital, I like to use my genital, I have no problem with it because I do not feel less of a woman. And that it will not be a surgery that will guarantee me womanhood”* (3-F.F.BR).

“*In our country and the rest of the world, this will not change without education. The only way to end homophobia, transphobia, prejudice and discrimination is with school education”* (2-C.F.PT).

**Figure 1:**
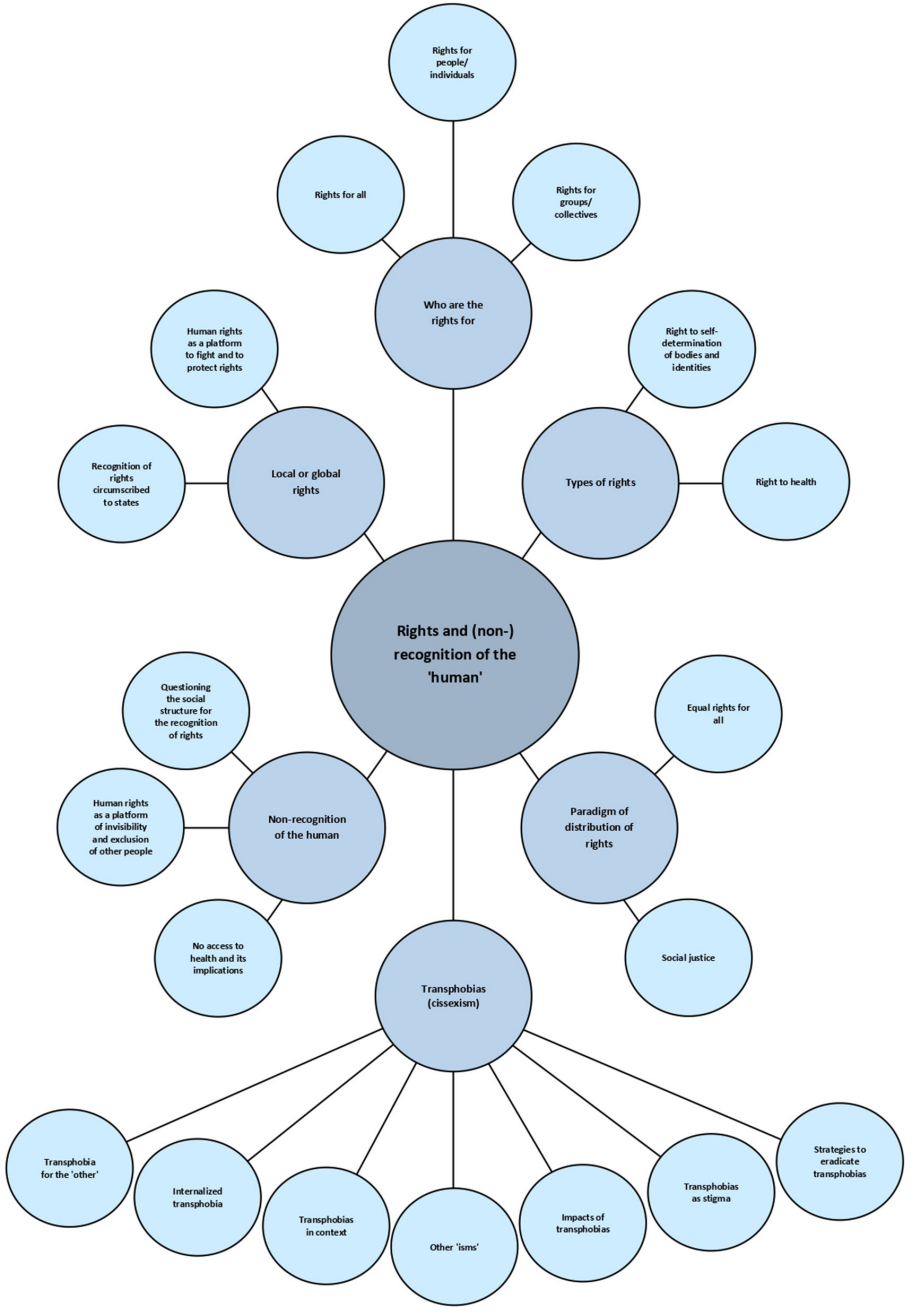
Thematic map of the analysis.

To better illustrate the central organizer “rights and (non-) recognition of the ‘human”' as well as the 6 themes of the analysis, Figure 1: thematic map of the analysis is presented.

## Conclusion

The discourses of participants show the way people (de)construct their identities is related to the way they think about transsexualities and their experiences. This occurs in an intersectional dimension with their historical, sociocultural, economic and political contexts creating matrices to understand gender. Thus, matrices permeate the (always diverse) expressions of their identities.

The analysis of the narratives allowed us to problematize that the legitimation of (dis)identity(s) should focus on people's self-definition and self-determination, in opposition to the attempt to legitimize any hetero-elected identity, specially the one that has been determined by the biomedical model.

Through the study, we can reinforce that trans people are heterogeneous and, as such, have differences at the economic, ideological, geographic, and social levels. These differences shape the various ways trans people conceptualize their own identities and transsexualities. This heterogeneity is also present in how different people position themselves in relation to the medical model.

This study shows that some trans people perform body modifications in order to combat the transphobia/cissexism they have experienced throughout their life paths. In addition, they believe that by making their bodies conforming they will be more desirable and they will be able to desire. The cisnormativity process occurs through the idea, present in the narratives of some people, that their bodies conformity will result in less discrimination and, therefore, will allow them more satisfactory experiences of their personal and social identities.

Based on the narratives of participants, the right to health is also problematized and it is concluded that should be no requirement related to the existence of any suffering or maladjustment. This access should only be based on the right to access health, broadening the very concept of health of the World Health Organization (2010) and, therefore, promoting the persons' wellbeing.

In this study, it is also recognized that rights have been circumscribed to certain international, regional and/or national contexts. The participants of the study pointed out that rights are local, not global, and that they are dependent on the legislation in force in each country, which is influenced by regional and international law.

Furthermore, they mentioned that human rights can also be understood as a platform for invisibility and exclusion of others. Although the participants recognize that the human rights platform still serves as a tool to fight and to protect rights, they also emphasize that it continues to erase and exclude groups that, by not being referred, have not been recognized as rights holders.

The study also allows us to rethink the violence that is exercised on trans people as a continuum, whether through “normalization devices” by medical, family, public space contexts, or even through internalized transphobia. While social structures produce and sustain transphobias, the same structures are responsible for combating them by changing the paradigm about the conception of transsexualities.

Transversally, the epistemologies adopted in this work, as well as the discourses that shaped it, justify the need to recognize the plurality of identity subjectivation processes. Therefore, it is a matter of conceiving and affirming transsexuality(ies) as non-pathological processes; of highlighting the importance of adopting human rights as a platform for the recognition of rights, considering that if the same platform does not attend to the specificities of groups, it will continue to reproduce processes of exclusion; of reinforcing the intersection of the various oppressions; of highlighting the importance of recognizing the self-determination of identities and adopting the proposals of depathologizing transsexualities; and of keeping in mind that the contexts of support/alliance are crucial for the wellbeing of people who do not conform to gender norms.

It is important to problematize, within the critique of the current medical model, that sexual reassignment surgery may not be an intended goal for some trans people (for reasons of, for example, health, implications of a surgical intervention, fear of loss of pleasure, among others). In some cases, trans people undergo the surgeries so that they can match their sex to their gender. However, this desire has another underlying: that the surgeries serve as a reparative tool for the recognition of their belonging to humanity. Instead of sexual reassignment surgery, for these trans people, the legal recognition of their sex and identity would be the most appropriate for their physical and psychological wellbeing (Garaizabal, 2010; Schramm et al., 2011).

From a human rights perspective, the pathologization of transsexuality is contrary to the right to free expression of gender identity and the right to free access to health care, rights recognized as inalienable (e.g., Corrêa and Muntarbhorn, 2007; Hammarberg, 2010; Suess, 2010; Ramos, 2011; UN, 2011; Hidalgo, 2022).

Critical human rights perspectives include the rights of all people, recognizing their identity belonging to specific axes of oppression. This inclusion of people considering their belonging is a characteristic of a real pluralization project of democratic societies (Schritzmeyer, 2008; Schramm et al., 2011).

Based on our work, we can say that for a liberating and emancipatory human rights project, it is important to train professionals who interact and/or work with this population concerning the diversity of trans life trajectories (Moita, 2001, 2006; Bailey et al., 2022), raising awareness about the full respect for trans identities. These professionals play a key role and should help these people to fully live an identity and a body culturally understood as corresponding to a particular gender, if they so wish (Miguel et al., 2008; Pinto and Moleiro, 2012).

Non-governmental organizations—working with the LGBTQI+ population, with trans people only and/or with issues related to sexual and gender oppression—should also contribute to social change and stimulate the development of a critical human rights perspective related to identities, genders and sexualities (Garaizabal, 2010; Alves, 2012).

In addition to the depathologization of trans identities and legislative changes at various levels of law (international, regional and national), it is important that these changes to combat transphobia/cissexism are carried out with an ethical sense of positive value about gender diversity (Stryker, 2013).

To conclude it is essential that the fight against transphobia and other forms of discrimination occurs in a dynamic and systemic relationship at three levels: at the macro-political level, through the approval of laws by the State and international bodies; through mesopolitics, from the policies of institutions that recognize and value diversity; and, through micropolitics, in the concrete actions of people's lives.

The collected and analyzed data reinforce heterogeneity, so it becomes essential to assume respect for this plurality if we intend to re/know the dignity of these people.

## Data availability statement

The datasets presented in this article are not available for ethical reasons of anonymity and confidentiality of the people interviewed.

## Ethics statement

Ethical review and approval was not required for the study on human participants in accordance with the local legislation and institutional requirements. The participants interviewed were all adults (over 18 years old) and provided their written informed consent to participate in this study.

## Author contributions

LR: conceptualization, data curation, formal analysis, funding acquisition, investigation, methodology, project administration, resources, software, and roles/writing—original draft. NSC and CN: supervision. LR and ARP: writing—review, translation, and editing. All authors contributed to the article and approved the submitted version.

## Footnotes

^1^Transphobia has been defined as a form of discrimination, violence, hatred and rejection against trans people or those who break with socially imposed gender norms (Jesus, 2012; Platero, 2014). In addition, it has also been conceptualized as a form of oppression for all people due to gender impositions, which results in: oppression of trans people by society; oppression of trans people by other trans people; and/or oppression of oneself for being trans, which is commonly referred to as “internalized transphobia” (Lewis and Arnold, 1998; Platero, 2014). Internalized transphobia can also be defined as the impact of discrimination on the way trans subjectivities are constructed (Missé, 2014). In the case of Gisberta, despite the different ongoing marginalization the crime practice was highly discussed and the court disregarded the transphobic hate component. It was only due to the action of LGBT activism movement that Europe pressured the Portuguese State to recognize the crime as based in aggravating factor of transphobia.

^2^Regarding suicide, when historically oppressed people commit suicide due to their nonconforming gender identity, what is at stake is the transphobic society that does not recognize diversity and adds vulnerability to people's lives. Here, Lara's experiences put her in a situation of self-hatred, also known as internalized transphobia. However, that self-hatred was not Lara's responsibility, but the responsibility of a society that is transphobic.

^3^Law No. 7/2011 de 15 de março (2011). *Cria o procedimento de mudança de sexo e de nome próprio no registo civil e procede à décima sétima alteração ao Código do Registo Civil*. Lisboa: Diário da República, 1ª série – N° 52, 1450–1451. Available online at: http://dre.pt/pdf1sdip/2011/03/05200/0145001451.pdf.

^4^Law No. 322-A/2001 de 14 de dezembro. Aprova o Regulamento Emolumentar dos Registos e Notariado. Lisboa: Diário da República n.° 288/2001, 1° suplemento, série I-A de 2001-12-14, 2-12. Available online at: https://dre.pt/dre/detalhe/decreto-lei/322-a-2001-330632.

^5^Law No. 19/2013 de 21 de fevereiro (2013). *procede à 29*.ª *alteração ao Código Penal, aprovado pelo Decreto-Lei n*.° *400/82, de 23 de setembro, e primeira alteração à Lei n*.° *112/2009, de 16 de setembro, que estabelece o regime jur*í*dico aplicável à prevenção da violência doméstica, à proteção e à assistência das suas v*í*timas*. Diário da República, 1.ª série, N.° 37. Available online at: https://dre.pt/application/dir/pdf1sdip/2013/02/03700/0109601098.pdf.

^6^Law No. 38/2018 de 7 de agosto (2018). *Direito à autodeterminação da identidade de género e expressão de género e à proteção das caracter*í*sticas sexuais de cada pessoa*. Lisboa: Diário da República, 1ª série – N° 151, 3922–3924. Available online at: https://dre.pt/dre/detalhe/lei/38-2018-115933863.

^7^Order No. 7247/2019 de 16 de agosto (2019). *Estabelece as medidas administrativas para implementação do previsto no n*.° *1 do artigo 12*.° *da Lei n*.° *38/2018, de 7 de agosto*. Presidência do Conselho de Ministros e Educação - Gabinetes da Secretária de Estado para a Cidadania e a Igualdade e do Secretário de Estado da Educação. Lisboa: Diário da República, 2ª Série – N° 156, 21–23. Available online at: https://dre.pt/dre/detalhe/despacho/7247-2019-123962165.

^8^Ordinance No. 2,803, de 19 de novembro (2013). *Redefine e amplia o Processo Transexualizador no Sistema Único de Saúde (SUS)*. Ministério da Saúde, Gabinete do Ministro. Available online at: http://bvsms.saude.gov.br/bvs/saudelegis/gm/2013/prt2803_19_11_2013.html.

^9^Ordinance No. 73 de 28/06/2018 (2018). *Dispõe sobre a averbação da alteração do prenome e do gênero nos assentos de nascimento e casamento de pessoa transgênero no Registro Civil das Pessoas Naturais (RCPN)*. Available online at: https://atos.cnj.jus.br/atos/detalhar/2623.

^10^Transvestites is a designation used in the Brazilian context. This is an opposite designation to those called “transvestite,” “transvestism,” or “cross-dresser” in the European context and, specifically, in Portugal. In Portugal, “transvestite” refers to people who sporadically dress and express an opposite gender to the one assigned at birth, but who tend to identify with the sex they were assigned at birth (Missé, 2014; Platero, 2014). In Brazil we can understand transvestites as people who self-identify as women and often change characteristics in their body to conform to their gender but do not alter their genitals.

## References

[B1] Alves H. (2012). Introdução ao Transfeminismo. Transfeminismo. Available online at: https://transfeminismo.com/introducao-ao-transfeminismo/

[B2] Arán M. Murta D. (2009). Do diagnóstico de transtorno de identidade de género às redescrições da experiência da transexualidade: Uma reflexão sobre género, tecnologia e saúde. Phys. Rev. Saúde Coletiva 19, 15–41. 10.1590/S0103-73312009000100003

[B3] Bailey D. Calasanti T. Crowe A. di Lorito C. Hogan P. de Vries B. (2022). Equal but different! Improving care for older LGBT+ adults. Age Ageing 51, 1–7. 10.1093/ageing/afac142 35751872

[B4] Braun V. Clarke V. (2006). Using thematic analysis in psychology. Qual. Res. Psychol. 3, 77–101. 10.1191/1478088706qp063oa

[B5] Braun V. Clarke V. (2013). Successful Qualitative Research: A Practical Guide for Beginners. Washington, DC: SAGE Publications.

[B6] Butler J. (1999). Gender Trouble: Feminism and the Subversion of Identity. New York, NY: Routledge.

[B7] Butler J. (2004). Undoing Gender. New York, NY: Routledge.

[B8] Cabezas L. P. Ortega E. Galán J. I. P. (2013). “Adolescentes transexuales en las aulas. Aproximación cualitativa y propuestas de intervención desde la perspectiva antropológica,” in Transexualidad, Adolescências y Educación: Miradas Multidisciplinares, eds O. Cabrera, and L. Cabezas (Madrid: Egales Editorial), 189–216.

[B9] Cohen-Kettenis P. Owen A. Kaijser V. Bradley S. Zucker K. (2003). Demographic characteristics, social competence, and behavior problems in children with gender identity disorder: a cross-national, cross-clinic comparative analysis. J. Abnorm. Child Psychol. 31, 41–53. 10.1023/A:1021769215342 12597698

[B10] Coll-Planas G. (2010). “Indroducción” in El género desordenado: Críticas en torno a la patologización de la transexualidad, eds M. Missé, and G. Coll-Planas (Barcelona: Egales), 15–25.

[B11] Corrêa S. Muntarbhorn V. (2007). The Yogyakarta Principles: Principles on the Application of International Human Rights Law in Relation to Sexual Orientation and Gender Identity. Available online at: https://yogyakartaprinciples.org/

[B12] Costa C. Pereira M. Oliveira J. M. Nogueira C. (2010). “Imagens sociais das pessoas LGBT” in Estudo sobre a discriminação em função da orientação sexual e da identidade de género, eds C. Nogueira and J. M. Oliveira (Lisboa: CIG), 93–147.

[B13] Creighton A. Kivel P. (1992). Helping Teens Stop Violence: A Practical Guide for Counselors, Educators, and Parents. Long Beach, CA: Hunter House CA.

[B14] Fernández-Rouco N. Sánchez F. González R. (2012). Transexualidad y vejez: una realidad por conocer. Rev. Kairós Gerontol. 15, 15–25. 10.23925/2176-901X.2012v15i3p15-25

[B15] Garaizabal C. (2010). “Transexualidades, identidades e feminismos,” in El género desordenado: Críticas en torno a la patologización de la transexualidad, eds M. Missé, and G. Coll-Planas (Barcelona: Egales), 125–140.

[B16] Hammarberg T. (2010). Derechos humanos e identidad de género. Available online at: https://transrespect.org/wp-content/uploads/2015/08/Hberg_es.pdf

[B17] Hidalgo S. L. (2022). Trans rights: the ongoing debate in Latin American legal agendas. Age Hum. Rights J. 18, 163–180. 10.17561/tahrj.v18.7061

[B18] Jesus J. G. (2012). Orientações sobre identidade de gênero: conceitos e termos. Guia técnico sobre pessoas transexuais, travestis e demais transgêneros, para formadores de opinião. Available online at: https://www.diversidadesexual.com.br/wp-content/uploads/2013/04/GÊNERO-CONCEITOS-E-TERMOS.pdf

[B19] Jesus J. G. Alves H. (2010). Feminismo transgênero e movimentos de mulheres transexuais. Cronos 11, 8–19.

[B20] Kapur R. (2006). Human rights in the 21st century: take a walk on the dark side. Sydney Law Rev. 28, 664–687. 10.4324/9780203814031

[B21] Kymlicka W. (1995). Multicultural Citizenship: A Liberal Theory of Monority Rights. New York, NY: Oxford University Press.

[B22] Kymlicka W. (2001). Politics in the Vernacular: Nationalism, Multiculturalism and Citizenship. Kingston, ON: Queen's University.

[B23] Lewis J. Arnold M. (1998). “From multiculturalism to social action” in Social Action: A mandate for Counselors, eds C. C. Lee, and G. R. Walz (Alexandria, VA: American Counseling Association and ERIC/CASS.Lopes), 51–66.

[B24] Lopes F. H. (2015). “Agora, as mulheres são outras: Travestilidade e envelhecimento” in Transfeminismo: Teorias e Práticas, ed. J. G. Jesus (Rio de Janeiro: Metanoia Editora), 171–192.

[B25] Madson N. H. (2022). I am an ordinary citizen: human rights discourse and the limits of human rights law. Am. Anthropol. 124, 504–514. 10.1111/aman.13737

[B26] Miguel T. B. Vierge S. A. Schaffer I. G. Ansio F. G. Montenegro J. L. Antonio I. E.. (2008). Una refleción sobre el concepto de género alredor de la transexualidad. Rev. Asoc. Esp. Neuropsiquiatr. 28, 211–226. 10.4321/S0211-57352008000100013

[B27] Missé M. (2014). Transexualidades: Outras Miradas Posibles. Madrid: Egales Editorial.

[B28] Moita G. (2001). Discursos sobre a homossexualidade no contexto clí*nico: A homossexualidade de dois lados do espelho* (dissertation). Instituto de Ciências Biomédicas de Abel Salazar da Universidade do Porto: Universidade do Porto.

[B29] Moita G. (2006). A patologização da diversidade sexual: Homofobia no discurso de clínicos. Rev. Crítica Ciênc. Soc. 76, 53–72. 10.4000/rccs.862

[B30] Mullally J. (2009). Review essay feminist reconstructions of universalism and the discourse of human rights. Int. J. Law Context 5, 87–92. 10.1017/S1744552309005059

[B31] Oliveira A. L. (2015). “Os homens transexuais brasileiros e o discurso pela (des)patologização das identidades (trans),” in Transfeminismo: Teorias e Práticas, ed J. G. Jesus (Rio de Janeiro: Metanoia Editora), 102–119.

[B32] Panteras Rosa (2006). A vergonha que se esperava. Available oinline at: http://panterasrosa.blogspot.com/2006/07/

[B33] Parker I. (1998). Social Constructionism, Discourse and Realism. London: Routledge.

[B34] Pearce R. (2018). Understanding Trans Health: Discourse, Power and Possibility. Bristol: Policy Press.

[B35] Pereira M. M. (2012). Fazendo Género no Recreio: A Negociação do Género em Espaço Escolar. Lisboa: ICS.

[B36] Pillay N. (2013). Nascidos livres e iguais: orientação Sexual e Identidade de Género no Regime Internacional de Direitos Humanos. Available online at: https://www.ohchr.org/sites/default/files/BornFreeAndEqualLowRes_Portuguese.pdf

[B37] Piñeroba J. (2008). Transexualidad, intersexualidad y dualidade de género. Barcelona: edicions bellaterra.

[B38] Pinto N. Moleiro C. (2012). As experiências dos cuidados de saúde de pessoas transexuais em Portugal: perspetivas de profissionais de saúde e utentes. Psicologia 26, 129–151. 10.17575/rpsicol.v26i1.266

[B39] Platero R. L. (2014). Trans^*^*exualidades: Acompañamiento, factores de salud y recursos educativos*. Barcelona: edicions bellaterra.

[B40] Prilleltensky I. Fox D. (1997). “Introducing critical psychology: values, assumptions, and the status quo,” in Critical Psychology: An Introduction, eds D. Fox, and I. Prilleltensky (London: SAGE Publications), 3–20.

[B41] Ramos M. (2011). Transgender Persons and Family Life: The issues of Sterilisation and Loss of Child Custody Rights (European Master's Degree in Human Rights and Democratisation). Lund University, Lund, Sweden.

[B42] Roberts A. Rosario M. Corliss H. Koenen K. Austin B. (2012). Childhood gender nonconformity: a risk indicator for childhood abuse and posttraumatic stress in youth. Pediatrics 129, 410–417. 10.1542/peds.2011-1804 22351893 PMC3289524

[B43] Rodrigues J. W. C. Barbosa B. R. S. N. Siva L. V. (2021a). Combating transfobia in the public safety policy agenda in Brazil: current scenario and challenges. J. Inst. Stud. 7, 1060–1080. 10.21783/rei.v7i3.490

[B44] Rodrigues L. (2016). Viagens Trans(Género) em Portugal e no Brasil: Uma Aproximação Psicológica Feminista Crí*tica* (Doctoral thesis). Universidade do Porto, Porto, Portugal.

[B45] Rodrigues L. Carneiro N. S. Nogueira C. (2021b). “Corpos das/nas Margens e Vidas Vulnerabilizadas: Envelhecimento de Pessoas Trans,” in Envelhecimento, Género e Sexualidades, eds S. I. Magalhães, and C. Nogueira (Coleção Debater o Social, 55. V. N. Famalicão: Edições Húmus), 241–266.

[B46] Romboli S. (2021). The right to sexual identity in the interpretation of the european court of human rights: Between the national margin of appreciation and the creation of common rules. Rev. Catalana Dret Públic 63, 231–249. 10.2436/rcdp.i63.2021.3684

[B47] Santos B. S. (1997). Por uma concepção multicultural de direitos humanos. Rev Crit Ciências Soc. 48, 11–32.

[B48] Santos G. S. (2009). “Rompendo o silêncio e a invisibilidade: lésbicas negras de Salvador” in Anais do Seminário Internacional Enlaçando Sexualidades - Educação, Saúde, Movimentos Sociais, Direitos Sexuais e Direitos Reprodutivos (Salvador).

[B49] Schramm F. Barboza H. Guimarães A. (2011). A moralidade da transexualidade: aspectos bioéticos e jurídicos. Rev. Redbioética UNESCO 1, 66–77.

[B50] Schritzmeyer A. L. P. (2008). “A defesa dos direitos humanos é uma forma de “ocidentalcentrismo”?” in *Anais da 26*^a^ *Reunião Brasileira de Antropologia - Direitos Humanos, Práticas de Justiça e Diversidade Cultural* (Porto Seguro).

[B51] Sennott S. (2011). Gender disorder as gender oppression: a transfeminist approach to rethinking the pathologization of gender non-conformity. Women Ther. 34, 93–113. 10.1080/02703149.2010.532683

[B52] Stryker S. (2013). “Prefacio” in Transrespeto versus Transfobia en el Mundo: un studio Comparativo de la situación de los derechos humanos de las personas Trans, eds C. Balzer, and J. S. Hutta (Serie de publicaciones de tvt, 7, Transgender Europe), 12–17. Available online at: https://transrespect.org/wp-content/uploads/2015/08/TvT_research-report_ES_.pdf

[B53] Suess A. (2010). “Análisis del panorama discursive alrededor de la despatologización trans: procesos de transformación de los marcos interpretativos en diferentes campos sociales” in El género desordenado: Críticas en torno a la patologización de la transexualidad, eds M. Missé, and G. Coll-Planas (Barcelona: Egales), 29–54.

[B54] Suess A. (2011). Despatologización trans y práctica arteterapéutica. Arte Políticas Identidade 4, 107–126.

[B55] TGEU (2015a). Trans Murder Monitoring 2015. Available online at: https://transrespect.org/en/transgender-europe-idahot-tmm-2015/

[B56] TGEU (2015b). Alarming Figures: Over 1,700 Trans People Killed in the Last 7 Years. Available online at: https://tgeu.org/tmm-idahot-update-2015/

[B57] TGEU (2021). TVT TMM Update • *Trans Day of Remembrance 2021: 375 Trans and Gender-Diverse People Reported Murdered in the Past Year*. Available online at: https://transrespect.org/en/tmm-update-tdor-2021/

[B58] Turner L. Whittle S. Combs R. (2009). Transphobic Hate Crime in the European Union. Available online at: https://www.ilga-europe.org/sites/default/files/transphobic_hate_crime_in_the_european_union_0.pdf

[B59] UN (2008). Declaração n° *A/63/635: Direitos humanos, orientação sexual e identidade de género*. Available online at: http://www.rio.rj.gov.br/dlstatic/10112/6767039/4186804/DeclaracaoA_63_635ONU.pdf

[B60] UN (2011). Leyes y prácticas discriminatorias y actos de violencia cometidos contra personas por su orientación sexual e identidad de género (A/HRC/19/41). Informe anual del Alto Comisionado de las Naciones Unidas para los Derechos Humanos e informes de la Oficina del Alto Comisionado y del Secretario General. Available online at: https://www.ohchr.org/sites/default/files/Documents/Issues/Discrimination/A.HRC.19.41_Spanish.pdf

[B61] Waite C. B. (1887). Suffrage a right of citizenship. Law J Library 1, 240–251.

[B62] Whittle S. (2006). “Foreword,” in The Transgender Studies Reader, eds S. Stryker, and S. Whittle (New York, NY: Routledge), xi-xvi.

[B63] Witten T. M. (2004). Life course analysis: the courage to search for something more: middle adulthood issues in the transgender and intersex community. J. Human Behav. Soc. Environ. 8, 189–224. 10.1300/J137v08n02_12

[B64] World Health Organization (2010). ICD-10: International Statistical Classification of Disease and Related Health Problems. Available online at: https://icd.who.int/browse10/2010/en

[B65] Zinkunegi A. A. (2013). “Transfronteras: un nuevo activismo mundial por la despatologización trans,” in Transexualidad, adolescencias y educación: miradas multidisciplinares, eds O. Cabrera, and L. Cabezas (Barcelona: Egales Editorial) 89–109.

[B66] Zucker K. Bradley S. (1995). Gender Identity Disorder and Psychosexual Problems in Children and Adolescents. New York, NY: Guilford Press. 10.1177/070674379003500603 2207982

